# Exploring the relationship between religiosity and coping strategies among individuals with mental illness in Uganda: a cross-sectional study

**DOI:** 10.1186/s40359-026-04291-1

**Published:** 2026-03-10

**Authors:** Joan Abaatyo, Godfrey Zari Rukundo, Samuel Maling, Emmanuel Alol

**Affiliations:** 1 Department of Psychiatry, Mulago Hospital, Kampala, Uganda; 2 Department of Psychiatry, King Ceasor University, Kampala, Uganda; 3 African Centre for Suicide Prevention and Research, Mbarara, Uganda; 4 Department of Psychiatry and Behavioural Neurosciences, McMaster University, Hamilton, ON Canada; 5 Department of Psychiatry, Faculty of Medicine, Mbarara University of Science and Technology, Mbarara, Uganda; 6 Department of Psychiatry, Faculty of Medicine, Lira University, Lira, Uganda

**Keywords:** Coping strategies, Mental illness, Religiosity, Uganda

## Abstract

**Background:**

Mental illnesses are leading causes of disability worldwide, and individuals often turn to religion as a source of support and resilience. In Uganda, where religious participation is deeply embedded in society, the influence of religiosity on coping strategies among individuals with mental illnesses remains underexplored. This study examined the relationship between religiosity and coping strategies among people with mental illnesses at Mbarara Regional Referral Hospital in southwestern Uganda.

**Methods:**

A cross-sectional study was conducted among 400 adult outpatients at Mbarara Regional Referral Hospital. Religiosity was assessed using the Duke University Religion Index (DUREL), and coping strategies were measured using the Brief Coping Orientation to Problems Experienced (Brief COPE) scale. Exploratory factor analysis was used to classify coping strategies into positive and negative. Multivariable logistic regression examined associations between religiosity dimensions and positive coping while adjusting for socio-demographic and clinical factors.

**Results:**

Negative coping strategies were more prevalent (61.2%) than positive strategies (38.8%). In adjusted analyses, higher organizational religious activity was associated with increased odds of positive coping (adjusted odds ratio [aOR] = 1.32, 95% CI: 1.10 to 1.57), while higher non-organizational religious activity was associated with reduced odds of positive coping (aOR = 0.69, 95% CI: 0.60 to 0.81). Family history of mental illness (aOR = 1.67, 95% CI: 1.06 to 2.26) and a diagnosis of bipolar disorder (aOR = 1.95, 95% CI: 1.01 to 3.76) were also associated with greater use of positive coping.

**Conclusion:**

Organizational religious activity is independently associated with adaptive coping among individuals with mental illness, whereas non-organizational religious activity is linked to reduced likelihood of positive coping. The high reliance on maladaptive coping strategies underscores the need for comprehensive, culturally sensitive mental health interventions that integrate psychosocial and faith-based support. Further longitudinal research is needed to clarify causal pathways.

## Introduction

Mental illnesses are leading causes of disability globally and are often accompanied by profound emotional distress and functional impairment [1]. Many individuals living with mental illness seek ways to manage this distress by drawing on external sources of support, including religion and spirituality [2]. Religion encompasses organized beliefs, practices, and communal structures that may shape how individuals interpret suffering, seek meaning, and respond to psychological challenges [3]. As such, religiosity has increasingly been recognised as a potentially important resource for coping with mental health difficulties [4].

Existing literature suggests that higher levels of religiosity are associated with more favorable mental health outcomes, including lower levels of hopelessness and anxiety, as well as better overall psychological adjustment [4]. In addition, religiosity has been linked to behavioral and emotional outcomes, such as reduced substance use and a lower risk of suicidal behaviour [5–8]. However, much of this evidence originates from high-income countries and is largely based on community or general population samples rather than individuals with clinically diagnosed mental disorders [5, 6, 9]. The extent to which these findings are applicable to low-resource settings remains uncertain, particularly in contexts where access to formal mental health services is limited and religious institutions often play a central social and supportive role [10].

Religiosity is a multidimensional construct that includes organisational religious activity (ORA), non-organisational religious activity (NORA), and intrinsic religiosity (IR), each of which may relate differently to mental wellbeing and coping [8, 11–13]. Coping strategies, defined as the cognitive and behavioural efforts individuals use to manage internal or external stressors, may be adaptive (e.g. problem-focused or emotion-focused coping) or maladaptive (e.g. avoidant coping) [14]. Prior studies indicate that the relationship between religiosity and coping is complex and may be influenced by socio-demographic factors such as age, gender, and education; social factors including social support and cultural norms; and clinical factors such as illness severity and treatment context, particularly in mental health conditions such as depression, anxiety disorders, psychotic disorders, and substance use disorders [15–17]. Specifically, organizational religiosity has been shown to facilitate adaptive coping through access to social support and collective meaning-making [18], whereas private religious practices may show mixed associations depending on whether they reflect active spiritual engagement or passive forms of coping such as deferral or avoidance [19]. However, these associations have been insufficiently explored among psychiatric populations in low-income and low-resource settings.

In Uganda and similar sub-Saharan African contexts, religion is deeply embedded in everyday life and often serves as a primary source of emotional, social, and moral support [20–25]. Despite this, there is a paucity of empirical research examining how different dimensions of religiosity are associated with coping strategies among individuals receiving psychiatric care, including clinically diagnosed psychiatric outpatients. Most available studies focus on general populations or rely heavily on evidence from high-income countries, limiting their relevance to clinical populations in low-resource health systems. This study is informed by by Lazarus and Folkman’s transactional theory of stress and Coping Theory [26], which conceptualizes coping as cognitive and behavioural responses to stressors, and by Pargament’s religious coping framework [27], which distinguishes positive and negative forms of religious coping and emphasizes the role of religious communities and meaning-making. Given the central role of religion in Uganda and limited psychiatric service access, understanding how religiosity relates to coping among psychiatric patients is essential for culturally responsive care. This study therefore sought to address this gap by examining the association between religiosity and coping strategies among adults with clinically diagnosed mental disorders in Uganda. Understanding these relationships is important for informing culturally sensitive, recovery-oriented mental health care and for integrating patients’ existing coping resources into clinical practice. To guide the study, we posed the following research questions:

1. What is the relationship between different dimensions of religiosity (organizational, non-organizational, and intrinsic) and the use of positive or negative coping strategies among individuals with mental illness in Uganda?
2. Which socio-demographic and clinical factors are associated with the use of positive versus negative coping strategies among individuals with mental illness?
3. Which dimensions of religiosity predict greater use of faith-based coping mechanisms?

## Methods

### Study design and setting

This facility-based cross-sectional study design was conducted at the psychiatry ward of Mbarara Regional Referral Hospital (MRRH) located in Mbarara city, southwestern Uganda. The ward provides inpatient and outpatient mental health services, including pharmacological and psychological care. MRRH functions as the main referral facility for the Ankole sub-region and nearby areas in Western Uganda, serving an estimated population of 3 million. Its catchment area includes districts such as Mbarara, Bushenyi, Ntungamo, Kiruhura, Ibanda, Buhweju, Rubirizi, Mitooma, and Isingiro. Additionally, the hospital receives referrals from adjacent regions like Kabale and Masaka, and even from neighboring countries such as Rwanda, Democratic Republic of Congo, and Tanzania [28]. The study was conducted at a single regional referral hospital to ensure consistency in diagnostic and treatment practices and to enhance data quality. As a high-volume referral centre, MRRH serves a diverse population from multiple districts and neighbouring countries, offering broad clinical representation. This single-site approach was feasible and appropriate given the exploratory nature of the study and limited evidence from low-resource clinical settings.

### Study population and study procedure

#### Study population

The study population comprised adults (≥ 18 years) with a documented diagnosis of a mental disorder who attended the outpatient psychiatry clinic at MRRH between April and June 2023.

Psychiatric diagnoses (e.g. schizophrenia, bipolar disorder, depressive disorders) were obtained from participants’ medical records and had been made during routine clinical care by psychiatry residents, qualified psychiatrists or psychiatric clinical officers. Diagnostic assessments followed standard clinical practice consistent with the Diagnostic and Statistical Manual of Mental Disorders, Fifth Edition (DSM-5) [29]. Diagnoses were based on comprehensive clinical interviews and mental status examinations as documented in the treatment charts. No structured diagnostic interviews were administered for the purposes of this study, as diagnoses were not the primary outcome but were used to characterise the clinical sample.

#### Study participants, sampling techniques and recruitment procedures

Eligible participants were adults aged 18 years and above with a documented psychiatric diagnosis who attended outpatient follow-up at MRRH during the study period, were clinically stable, and able to provide informed consent. Individuals who were acutely psychotic, severely cognitively impaired, medically unstable, or unable to comprehend the study procedures were excluded. Participants were recruited using consecutive sampling, whereby all eligible patients attending the clinic were approached until the required sample size was achieved. Two trained research assistants conducted recruitment after clinic consultations under supervision of the principal investigator.

#### Sample size calculation

Although the exact number of eligible patients during the study period was not formally documented, clinic records indicated an average attendance of 10–15 patients per day. Over the three-month data collection period, this corresponded to an estimated population of 600–900 individuals. The sample size was calculated using the Kish–Leslie formula for cross-sectional studies [30]:

$$n=\frac{{Z}^{2}p(1-p)}{{d}^{2}}$$

where *n* represents the required sample size, *Z* denotes the standard normal critical value corresponding to a 95% confidence level (*Z* = 1.96), *p* is the estimated prevalence of the outcome, and *d* is the specified margin of error (5%). A prevalence of 50% was assumed to maximise the sample size in the absence of prior local prevalence estimates for religiosity-related coping among psychiatric populations. The calculated minimum sample size was adjusted for a 10% non-response rate, resulting in a final target sample of 400 participants, who were enrolled consecutively.

The Kish–Leslie formula is mathematically equivalent to Cochran’s formula for large populations and is widely used in epidemiological studies in low- and middle-income settings due to its simplicity and applicability in facility-based surveys [30].

### Study variables

#### Dependent variable

The primary dependent variable was coping strategies, operationalised using the Brief Coping Orientation to Problems Experienced (Brief COPE) Inventory [15] and analysed as positive (adaptive) coping and negative (maladaptive) coping styles.

#### Independent variables

The main independent variables were dimensions of religiosity, assessed using the Duke University Religion Index (DUREL) [31].

#### Covariates and potential confounders

Socio-demographic variables considered as potential confounders included age, sex, marital status, level of education, employment status, and family history of mental illness. Clinical variables included psychiatric diagnosis as documented in the treatment chart, duration of mental illness, presence of chronic physical conditions, and family history of suicidal behaviour.

### Data collection and materials

#### Socio-demographic and clinical data

Data were collected in English and Runyankole–Rukiga (the predominant local language). Standard forward translation and back-translation procedures were used for the DUREL and Brief COPE, and discrepancies were resolved by consensus among bilingual mental health professionals. Research assistants were trained to administer the tools consistently. Socio-demographic and clinical information was collected using a structured researcher-administered questionnaire developed for this study. Clinical variables were verified from participants’ medical records to enhance accuracy.

#### Duke university religion index

Religiosity was evaluated using the DUREL, which assesses three core dimensions: ORA, NORA, and IR. ORA measures attendance at formal religious services, while NORA assesses time spent on private religious activities like prayer or meditation [32]. IR reflects how deeply individuals integrate religion into their daily lives, based on three items. ORA and NORA responses are rated on a 6-point scale (1: never to 6: several times a week), while IR responses use a 5-point scale (1: definitely not true to 5: definitely true). The mean score for each subscale was calculated to obtain ORA, NORA, and IR scores. An overall religiosity score was computed by combining the subscale scores. An internal consistency of this psychometric instrument was satisfactory (Cronbach’s alpha, α = 0.69).

#### Brief cope

Scores of the brief cope are presented for the two overarching coping styles: negative coping, which is characterized by the subscales of denial, substance use, venting, behavioral disengagement, self-distraction and self-blame. Positive coping is characterized by the subscales of active coping, positive reframing, planning, acceptance, seeking emotional support, and seeking informational support [15]. These 14 coping subscales are assessed with 28 questions. The responses to these questions are measured on a 4- point Likert-type scale with responses ranging from 1 (“I’ve not done this at all”) to 4 (“I’ve been doing this a lot”). The scores (ranging from 2 to 8) and the means for each coping method were then calculated. An internal consistency of this psychometric instrument was satisfactory (Cronbach’s alpha, α = 0.83).

### Data analysis

Data was analyzed using Stata version 15.0 (Stata Corp LLC, College Station, Texas, USA). Categorical variables were summarized using frequencies and percentages. Spearman’s rank correlation coefficients were used to examine unadjusted associations between dimensions of religiosity overall religiosity, ORA, NORA, and IR] and coping strategies, given the ordinal nature of the measures and the non-normal distribution of the data.

Exploratory factor analysis was performed on the 28 Brief COPE items, with the number of factors retained guided by the eigenvalue > 1 criterion and inspection of the scree plot. Data suitability for factor analysis was evaluated using the Kaiser–Meyer–Olkin (KMO) measure and Bartlett’s test of sphericity. Factors were extracted using principal axis factoring, which is appropriate for identifying latent constructs and does not require multivariate normality. To enhance interpretability, we applied an orthogonal varimax rotation. The final factor solution was selected based on both the eigenvalue threshold and the scree plot inflection point. An eight-factor solution was retained, as all items loaded meaningfully on a single factor (factor loadings ≥ 0.40) without substantial cross-loading. Model adequacy was supported by acceptable communalities and the proportion of variance explained (Appendix). Composite scores were calculated for each factor by summing item scores. For subsequent analyses, factor scores were dichotomized as < 1 (non-use of a coping strategy category) and ≥ 1 (use of a coping strategy category) to facilitate clinical interpretability and alignment with prior studies that classify coping strategies as present or absent. The eight coping factors were further grouped into positive (adaptive) and negative (maladaptive) coping strategies based on established literature [33, 34].

Chi-square tests were employed to analyze differences in coping strategies across the study characteristics. To determine factors associated with coping strategies, multiple logistic regression analyses were conducted based on prior statistical screening, with results reported as adjusted odds ratios (aORs) and corresponding 95% confidence intervals. Positive coping was treated as the outcome variable, and analyses examined its association with religiosity while adjusting for potential confounders. Multicollinearity was assessed using variance inflation factors (VIFs); total religiosity was excluded to avoid collinearity with ORA, NORA, and IR. Among diagnoses, Schizophrenia spectrum disorder” as the reference group because it has distinct clinical characteristics, including cognitive and functional impairments, that may influence coping differently compared to other disorders. No mediation or moderation analyses were conducted; covariates were included as potential confounders based on prior literature. A *p*-value of ≤ 0.05 was considered statistically significant, with 95% confidence intervals.

## Results

### Characteristics of study participants

A total of 400 participants were included in the analysis. The mean age was 38.6 years (Standard deviation (SD) = 13.1), with ages ranging from 18 to 83 years. The age distribution was slightly skewed to the right with the median age at 37 years and the interquartile range (IQR) between 29 and 47 years. The sample was almost evenly split, with 51.3% males (*n* = 205) and 48.8% females (*n* = 195). The majority were married or cohabited (42.5%, *n* = 170). Regarding clinical characteristics, 32% (*n* = 128) had lived with a mental disorder for more than 10 years, 26.8% (*n* = 107) for 5 to 10 years, 13.0% (*n* = 52) for 1 to 5 years, and 28.3% (*n* = 113) for less than a year. Bipolar disorder (BD) was the most frequently documented diagnosis (37.0%, *n* = 148), followed by schizophrenia spectrum disorders and substance use disorders (16.8%, *n* = 67), depression (16.5%, *n* = 66), HIV-related psychosis (7.8%, *n* = 31), and other diagnoses at 5.3% (*n* = 21).

### Levels of religiosity

The overall mean religiosity score was 12.0 (SD = 2.9), with ORA having the highest score (4.30, SD = 1.42), followed by IR (3.86, SD = 0.73) and then NORA (3.74, SD = 1.71). See Table 1.

**Table 1:** Levels of Religiosity and Coping Strategies Among Participants (*N* = 400)

Domain	Coping Category	Subdomain / Indicator	Mean (SD) / *n* (%)
Religiosity (DUREL)		ORA	4.30 (1.42)
		NORA	3.74 (1.71)
		IR	3.86 (0.73)
		Overall religiosity score	12.0 (2.9)
Overall Coping Strategies		Positive / Adaptive coping	155 (38.8)
		Negative / Maladaptive coping	245 (61.2)
Coping strategies	Positive coping	Emotional release and resolution	116 (29.0)
		Supportive problem solving	120 (30.0)
		Proactive coping	165 (41.2)
		Strategic reframing	28 (7.0)
		Divine comfort	164 (41.0)
		Humor and social venting	122 (30.5)
	Negative coping	Substance use and distraction	154 (38.5)
		Guilt-driven coping	127 (31.8)

*ORA *organisational religious activity,* NORA *non-organisational religious activity,* IR *intrinsic religiosity,* DUREL *Duke University Religion Index,* SD *Standard deviation,* % *percentage

### Distribution of coping strategies

Coping strategies, categorized into positive and negative based on factor analysis of the Brief COPE inventory, revealed that 61.2% (*n* = 245) of participants primarily used maladaptive strategies such as substance use, guilt, and behavioral disengagement, while 38.8% (*n* = 155) employed adaptive strategies including proactive coping, divine comfort, and problem-solving. Among adaptive strategies, proactive coping (41.2%) and divine comfort (41.0%) were the most frequently used. For maladaptive strategies, substance use, and distraction (38.5%) and guilt-driven coping (31.8%) were most prevalent (Table 1).

### Bivariate associations between participant characteristics and coping strategies

Participants who utilized adaptive coping strategies were statistically younger than those who utilized negative coping strategies i.e., mean of 37 vs. 40, respectively, p value = 0.030. Overall religiosity was higher among females than males (mean 12.4 vs. 11.4; *p* < 0.001), although coping strategy category did not significantly differ by sex (*p* = 0.362). There was no statistically significant difference across other study characteristics and coping strategies used. There was a small positive correlation between age and total religiosity score. For details, see Table 2.

**Table 2:** Bivariate associations of socio-demographic and clinical variables with coping strategies, alongside differences in overall religiosity across participant characteristics

Variable	Positive coping strategies *n* (%) 115 (38.8)	Negative coping strategies *n* (%) 254 (61.2)	t/X^2^ (*P*-value)	Overall religiosity *r*/mean (SD)	*p*-value
Age [*mean (SD)]*	37 (12.00)	40 (13.67)	-2.18 (0.030)	0.12	**0.020**
Sex					
Male	75 (36.59%)	130 (63.41%)	0.83 (0.362)	11.4 (3.2)	**< 0.001**
Female	80 (41.03%)	115 (58.97%)		12.4 (2.6)	
Marital status					
Separated/widowed/ divorced	35 (38.46%)	56 (61.54%)	1.39 (0.499)	12.0 (2.8)	0.158
Never married	59 (42.45%)	80 (57.55%)		11.5 (3.1)	
Married/Living with partner	61 (35.88%)	109 (64.12%)		12.1 (2.8)	
Employment status					
Employed	101 (36.07%)	179 (63.93%)	2.8 (0.093)	11.8 (2.9)	0.188
Unemployed	54 (45.00%)	66 (55.00%)		12.2 (3.0)	
Highest level of education					
Primary	59 (37.11%)	100 (62.89%)	1.48 (0.477)	11.8 (3.0)	0.441
Secondary	61 (42.66%)	82 (57.34%)		11.7 (3.1)	
Tertiary	35 (35.71%)	63 (64.29%)		12.2 (2.6)	
Duration of illness					
Less than a year	40 (35.40%)	73 (64.60%)	4.62 (0.202)	12.4 (2.6)	0.086
One to 5 years	20 (38.46%)	32 (61.54%)		12.3 (2.7)	
5 to 10years	36 (33.64%)	71 (66.36%)		11.8 (3.1)	
More than 10years	59 (46.09%)	69 (53.91%)		11.4 (3.1)	
Having a family history of mental illness					
No	71 (34.98%)	132 (65.02%)	2.47 (0.116)	11.9 (2.9)	0.911
Yes	84 (42.64%)	113 (57.36%)		11.9 (3.0)	
Diagnosis					
Schizophrenia spectrum disorder	25 (37.31%)	42 (62.69%)	7.13 (0.211)	12.0 (2.8)	0.341
Bipolar disorder	69 (46.62%)	79 (53.38%)		12.1 (2.9)	
Depression	22 (33.33%)	44 (66.67%)		11.9 (2.8)	
Substance use disorder	24 (35.82%)	43 (64.18%)		11.1 (3.3)	
HIV induced psychosis	9 (29.03%)	22 (70.97%)		12.1 (2.5)	
Others	6 (28.57%)	15 (71.43%)		11.9 (3.4)	
Chronic medical condition					
No	120 (39.34%)	185 (60.66%)	0.19 (0.662)	11.9 (3.0)	0.701
Yes	35 (36.84%)	60 (63.16%)		12.0 (2.9)	

*SD *Standard deviation,* % *percentage

*P*-value in bold are statistically significant at *p* < 0.05

### Correlations between religiosity dimensions and coping strategies

Correlation analyses examined unadjusted associations between dimensions of religiosity (ORA, NORA, IR) and specific coping strategies. Although several correlations were statistically significant, effect sizes were small (*r* = 0.10–0.40), indicating weak associations. ORA ORA showed a small positive correlation with divine comfort (*r* = 0.33), while NORA and IR were also weakly positively correlated with divine comfort (*r* = 0.27 and *r* = 0.26, respectively). Overall religiosity demonstrated weak positive correlations with proactive coping (*r* = 0.21) and divine comfort (*r* = 0.40), as well as a small positive correlation with substance use and distraction (*r* = 0.12). Negative coping showed weak negative correlations with ORA (*r* = − 0.13) and weak positive correlations with NORA (*r* = 0.18). Given the small magnitude of these coefficients, the findings were interpreted cautiously, reflecting subtle rather than strong relationships. Notably, divine comfort emerged as the coping strategy most consistently correlated with all dimensions of religiosity, suggesting that it may represent the primary faith-linked coping construct in this sample (Table 3).

**Table 3:** Correlation between coping strategies and religiosity

Variable	Religiosity *r*			
	Overall religiosity	ORA	NORA	IR
Negative coping	-0.05	-0.13*	0.18**	-0.06
Emotional release and resolution	-0.04	-0.12*	0.06	-0.11*
Substance use and distraction-based coping	0.12*	-0.12*	0.27**	-0.06
Supportive problem solving	0.08	-0.05	0.19**	-0.09
Proactive coping	0.21**	0.04	0.28**	0.01
Strategic reframing	-0.08	-0.02	-0.08	-0.05
Divine comfort	0.40**	0.33**	0.27**	0.26**
Guilt driven coping	-0.07	-0.10*	-0.02	-0.07
Humor and social venting	0.06	0.13*	0.03	-0.01

*ORA *organisational religious activity,* NORA *non-organisational religious activity, *IR *intrinsic religiosity

*=*p*-value < 0.05; **=*p*-value < 0.001; very high correlation positive (negative) = r^2^ = 0.90 to 1.00 (− 0.90 to − 1.00); high positive (negative) correlation = 0.70 to 0.90 (− 0.70 to − 0.90); moderate positive (negative) correlation = 0.50 to 0.70 (− 0.50 to − 0.70); low positive (negative) correlation = 0.30 to 0.50 (− 0.30 to − 0.50); Small correlation = 0.00 to 0.30 (0.00 to − 0.30)

*P*-value in bold are statistically significant

To account for multiple comparisons in the correlation analyses, a Bonferroni correction was applied. Given 36 correlation tests, the adjusted significance threshold was set at α = 0.0014.

### Multivariable analysis: religiosity as a predictor of coping strategies

Higher ORA was associated with increased likelihood of having positive coping strategies [adjusted odds ratio [aOR = 1.32, 95% confidence interval (CI) = 1.10 to 1.57, *p* = 0.002]. However, increase in NORA was associated with less likelihood of having positive coping strategies [aOR = 0.69, 95% CI (0.60 to 0.81), *p* < 0.001]. There was no relationship between intrinsic religiosity and coping strategies. Additionally, participants with a family history of mental illness had significantly higher odds of using positive coping strategies compared with those without such a history (aOR = 1.67, 95% CI: 1.06 to 2.26, *p* = 0.026). Similarly, individuals diagnosed with bipolar disorder were more likely to employ positive coping strategies than those with schizophrenia spectrum disorders, which served as the reference group (aOR = 1.95, 95% CI: 1.01 to 3.76, *p* = 0.047) (See Table 4).

**Table 4:** Logistic regression analysis for relationship between religiosity and coping strategies

Variable	Bi variable analysis	*p*-value	Multivariate analysis	*p*-value
	cOR (95% CI)		aOR (95% CI)	
ORA	1.12 (0.97 to 1.29)	0.126	1.32 (1.10 to 1.57)	0.002*
NORA	0.80 (0.71 to 0.90)	< 0.001	0.69 (0.60 to 0.81)	< 0.001**
IR	1.28 (0.96 to 1.71)	0.091	1.29 (0.93 to 1.79)	0.125
Age	0.98 (0.97 to 0.99)	0.031	0.99 (0.96 to 1.01)	0.279
Sex				
Male	1		1	
Female	1.20 (0.81 to 1.80)	0.362	1.24 (0.76 to 2.01)	0.391
Marital status				
Separated/widowed/ divorced	1		1	
Not married	1.18 (0.69 to 2.02)	0.548	0.93 (0.44 to 1.97)	0.851
Married/Living with partner	0.89 (0.53 to 1.51)	0.681	0.79 (0.43 to 1.46)	0.448
Employment status				
Employed	1		1	
Unemployed	1.45 (0.94 to 2.23)	0.094	1.47 (0.88 to 2.35)	0.141
Highest level of education				
Primary	1		1	
Secondary	1.26 (0.79 to 2.00)	0.325	1.10 (0.64 to 1.88)	0.731
Tertiary	0.94 (0.56 to 1.59)	0.822	0.87 (0.47 to 1.60)	0.653
Duration of mental illness				
Less than a year	1		1	
One to 5 years	1.14 (0.58 to 2.24)	0.704	1.18 (0.56 to 2.49)	0.665
5 to 10years	0.93 (0.53 to 1.61)	0.785	1.00 (0.54 to 1.87)	0.979
More than 10years	1.56 (0.93 to 2.62)	0.093	1.64 (0.88 to 3.08)	0.119
Having a family history of mental illness				
No	1		1	
Yes	1.38 (0.92 to 2.06)	0.116	1.67 (1.06 to 2.62)	0.026*
Diagnosis				
Schizophrenia spectrum disorder	1		1	
Bipolar disorder	1.46 (0.81 to 2.65)	0.204	1.95 (1.01 to 3.76)	0.047*
Depression	0.84 (0.41 to 1.71)	0.613	1.02 (0.47 to 2.23)	0.956
Substance use disorder	0.94 (0.47 to 1.90)	0.858	0.95 (0.44 to 2.07)	0.901
HIV induced psychosis	0.68 (0.27 to 1.72)	0.424	0.72 (0.23 to 2.21)	0.568
Others	0.67 (0.23 to 1.96)	0.466	0.65 (0.20 to 2.08)	0.468
Chronic medical condition				
No	1		1	
Yes	0.89 (0.56 to 1.45)	0.662	1.14 (0.62 to 2.11)	0.667

*ORA *organisational religious activity,* NORA *non-organisational religious activity,* IR *intrinsic religiosity

*: statistical significance at  *p* < 0.05; **: strong statistical significance at *p < 0.001 cOR *crude odd ratio*, aOR *adjusted odd ratio

*P*-value in bold are statistically significant

## Discussion

This study examined the association between different dimensions of religiosity and coping strategies among adults with mental illness in a low-resource setting in Uganda. The key findings were that ORA was the most used dimension of religiosity while negative coping strategies were more prevalent than positive coping strategies. ORA was independently associated with greater use of positive coping strategies, NORA was associated with reduced likelihood of positive coping, and IR showed no independent association with coping strategies. In addition, having a family history of mental illness and a diagnosis of BD were associated with increased use of positive coping strategies.

The higher mean score for ORA compared with IR and NORA indicates that communal and public expressions of religiosity were the most prominent form of religious engagement among participants in this Ugandan clinical sample. In Uganda, organized religious participation often extends beyond individual spirituality to encompass social support, shared meaning, moral guidance, and collective identity, which may be particularly salient for individuals living with mental illness who face stigma and social isolation [20–23]. From the Stress and Coping Theory proposed by Lazarus and Folkman, coping is shaped by individuals’ cognitive appraisal of stressors and the resources available to manage them. Within this framework, participation in organised religious communities may enhance perceived coping resources, such as social support, shared meaning, and collective problem-solving, thereby facilitating more adaptive responses to psychological distress [2, 26]. Consistent with this framework, divine comfort, defined as drawing emotional strength, reassurance, and meaning from faith, emerged as one of the most frequently used positive coping strategies in this study and showed the strongest correlations with organisational religious activity. This suggests that communal religious engagement may be particularly important for fostering faith-linked coping through shared worship, prayer, and collective meaning-making [35]. The comparatively lower scores for NORA may reflect the challenges of sustaining solitary religious practices in the context of mental illness, where symptoms such as low motivation or cognitive difficulties can limit private engagement, while IR alone may be insufficient to translate beliefs into effective coping without accompanying social or material resources [36].

The predominance of negative coping strategies, particularly substance use, distraction, and guilt-driven coping, observed in this study reflects the structural, social, and psychological constraints faced by individuals living with mental illness in Uganda. Similar patterns have been documented in other low-resource settings, where limited access to mental health services, persistent stigma, and economic hardship restrict opportunities for adaptive coping [37, 38]. Stigma further discourages help-seeking, pushing individuals toward self-management strategies that may be harmful [39, 40]. Additionally, cultural norms that normalize alcohol consumption [41] and a general lack of awareness about effective coping strategies contribute to this trend [42]. Distraction, while offering temporary relief, often prevents individuals from addressing underlying psychological distress [43]. Economic struggles, including poverty and unemployment, further limit access to healthier coping alternatives, reinforcing reliance on these negative strategies [42]. Addressing these issues requires improved mental health services, increased awareness, and interventions tailored to Uganda’s socio-cultural and economic context.

The finding that ORA was independently associated with greater use of positive coping strategies is consistent with Pargament’s Religious Coping Theory, which emphasises the role of positive religious coping, such as seeking spiritual support, collaborative problem-solving with faith communities, and meaning-making, in promoting psychological adjustment [44]. In this study, divine comfort represents a central expression of positive religious coping and appears to function as the primary pathway through which organisational religious activity supports adaptive coping. In the Ugandan context, organised religious participation provides access to structured social networks, moral guidance, emotional validation, and communal problem-solving, all of which may enhance perceived control and resilience [36, 45]. Prior studies report that public religious involvement is associated with adaptive coping and better mental health outcomes, highlighting the importance of the social embeddedness of religion in shaping coping behaviours [46]. Additionally, positive proactive coping strategies, such as seeking professional help, engaging in community support groups, or practicing self-care, are increasingly being adopted as mental health awareness grows in Uganda [35]. As individuals recognize the importance of taking charge of their well-being, proactive coping empowers them to seek help early, manage stress effectively, and build resilience, even in the face of stigma [35]. The integration of religious support with practical, action-oriented coping methods reflects a holistic approach to mental health in Uganda, where both spiritual and social resources are leveraged to improve well-being [44].

Although NORA correlated with some adaptive coping subdomains, the adjusted model showed that higher NORA was associated with reduced odds of overall positive coping, suggesting a complex or mixed relationship. The finding that higher levels of NORA were associated with a reduced likelihood of positive coping underscores the complexity of private religious practices and aligns with theoretical distinctions within religious coping frameworks. According to Pargament’s model, solitary religious practices may represent either active spiritual engagement or more passive coping styles, such as religious deferral, where individuals relinquish personal agency and rely exclusively on divine intervention [19, 44]. In such contexts, divine comfort derived from private religiosity may not be accompanied by the social reinforcement and practical support that appear critical for translating faith into adaptive coping behaviours [47]. In contexts characterised by limited external support, private religiosity may coexist with rumination, avoidance, or reliance on maladaptive coping strategies, including substance use [36]. Similar mixed or negative associations between solitary religious practices and coping have been reported in prior studies, particularly when such practices are not reinforced by communal support structures.

Although intrinsic religiosity (IR) was not independently associated with coping strategies in this study, contrary to some previous findings [10, 48], this result aligns with evidence indicating that deeply internalized religious beliefs do not consistently translate into adaptive coping behaviours in contexts characterized by structural adversity [49]. From the Stress and Coping Theory perspective, values and beliefs such as intrinsic religiosity may primarily influence cognitive appraisal of stress, but may be insufficient to shape behavioural coping in the absence of enabling social, economic, or health-system resources [26, 50, 51]. This may explain why intrinsic religiosity alone did not predict coping patterns in this sample.

Participants with a family history of mental illness were more likely to use positive coping strategies. This association may reflect increased mental health literacy, early exposure to coping resources, and greater normalisation of help-seeking within affected families [52]. Shared family experiences of managing mental illness can reduce stigma and foster adaptive coping through modelling, emotional support, and timely intervention [53]. Similarly, participants diagnosed with BD were more likely to use positive coping strategies compared with those with schizophrenia spectrum disorders. This is likely because individuals with BD often engage in long-term treatment, psychoeducation, and structured routines, particularly during euthymic periods, which may facilitate the development of adaptive coping strategies aimed at preventing relapse and maintaining stability [6, 54–57].

### Strengths and limitations

This study has several notable strengths. First, it examined multiple dimensions of religiosity rather than treating religiosity as a unidimensional construct, allowing for more nuanced interpretation. Second, the study focused on clinically diagnosed individuals receiving psychiatric care in a low-resource setting, addressing a major gap in the literature that is dominated by general population studies from high-income countries. Third, the use of validated psychometric instruments and multivariable analyses strengthened the robustness of the findings.

This study has several limitations that should be considered when interpreting the findings. Cross-sectional design prevents causal inferences between religiosity and coping strategies, and reliance on self-reported data introduces potential biases such as social desirability and recall bias. Because acutely unwell individuals were excluded, our findings primarily reflect coping patterns among clinically stable outpatients; coping profiles and religiosity–coping associations may differ in acute presentations, limiting generalizability to patients in crisis. As a facility-based, single-site study, the findings may have limited generalizability to broader psychiatric populations. Using consecutive sampling, selection bias is possible, particularly among patients not attending follow-up or those too unwell to participate; however, recruitment over a three-month period helped capture a diverse outpatient population. In addition, the use of consecutive sampling introduces potential selection bias, as individuals not attending outpatient follow-up or those who were acutely unwell and unable to participate may have been underrepresented. Although potential confounders were adjusted for, unmeasured factors like socioeconomic status and trauma history may have influenced the results. The study’s classification of coping strategies may oversimplify their complexity, and the DUREL might not fully capture the nuances of religious experiences in the Ugandan context. In addition, dichotomizing factor scores may have reduced variability and statistical power, potentially contributing to attenuation of observed associations. Furthermore, the modest internal consistency of the DUREL observed in this study may have introduced measurement error, potentially attenuating observed associations between religiosity and coping strategies. Additionally, the lack of clinical severity assessment and potential selection bias, excluding individuals in acute mental health crises, may have impacted the findings. Consequently, the findings mainly reflect coping patterns among clinically stable outpatients and may not be generalizable to individuals experiencing acute psychiatric episodes. While some correlations were statistically significant, their negligible strength raises questions about their practical significance. Finally, cultural and contextual influences on religious coping were not extensively explored, warranting further research into these variations.

### Implications of the study for practice, policy, and future research

The findings of this study have important implications for mental health practice, policy, and future research in low-resource settings. From a clinical and community practice perspective, the predominance of maladaptive coping strategies underscores the need for psychoeducation and skills-based that build adaptive coping, and integrated substance use prevention and treatment services within mental health care [58]. Strengthening collaborative referral pathways and support linkages between mental health services and faith communities may enhance social support, engagement, and acceptability of interventions by leveraging existing religious organizational structures, while ensuring that care remains grounded in evidence-based clinical practice [59, 60]. At the policy level, these findings highlight the importance of strengthening community mental health systems, expanding access to substance use prevention and treatment services, and embedding culturally sensitive mental health programs within primary healthcare and community settings, in line with global mental health recommendations for low- and middle-income countries [61, 62]. For future research, longitudinal and mixed-methods studies are needed to clarify causal pathways between religiosity and coping, explore the mechanisms through which communal religious engagement influences coping behaviors, and assess the effectiveness of culturally adapted interventions that integrate psychosocial and spiritual resources.

## Conclusions

This study highlights the complex relationship between religiosity and coping strategies among individuals with mental illness attending psychiatric care at Mbarara Regional Referral Hospital. Organizational religious activity was the most prominent dimension of religiosity and was independently associated with a higher likelihood of positive coping strategies, whereas non-organizational religious activity was associated with a reduced likelihood of positive coping, and intrinsic religiosity showed no independent association. Most individuals with mental illness relied on maladaptive coping strategies. In addition to religiosity, having a family history of mental illness and a diagnosis of bipolar disorder were associated with greater use of positive coping strategies. Collectively, these findings underscore the importance of comprehensive interventions that strengthen coping skills, address substance use, and leverage community-based support structures such as faith communities. Incorporating faith-sensitive approaches alongside structured psychosocial interventions may enhance the cultural relevance, acceptability, and effectiveness of mental health care in low-resource settings.

## Data Availability

The data supporting the findings of this study are available from the corresponding author upon reasonable request.

## Appendix

**Table:** Factor loadings of Brief COPE items across eight coping strategy factors

No.	Item	Factor1	Factor2	Factor3	Factor4	Factor5	Factor6	Factor7	Factor8
1	I’ve been turning to work or other activities to take my mind off things.	-0.111	-0.064	-0.115	**0.507**	-0.023	0.029	-0.040	0.044
2	I’ve been concentrating my efforts on doing something about the situation I’m in.	-0.027	-0.072	-0.057	**0.448**	-0.003	-0.037	0.006	-0.046
3	I’ve been saying to myself “this isn’t real.”	-0.049	0.123	0.262	-0.153	0.009	0.052	-0.163	-0.058
4	I’ve been using alcohol or other drugs to make myself feel better.	-0.016	-0.022	-0.014	0.006	**0.519**	-0.016	-0.003	-0.025
5	I’ve been getting emotional support from others.	0.309	-0.094	-0.051	-0.067	0.038	-0.059	-0.014	0.088
6	I’ve been giving up trying to deal with it.	-0.046	-0.100	**0.388**	-0.048	-0.066	0.024	0.056	0.006
7	I’ve been refusing to believe that it has happened.	0.036	0.031	0.076	0.159	0.033	-0.008	0.033	-0.113
8	I’ve been saying things to let my unpleasant feelings escape.	-0.085	-0.156	0.287	0.115	0.045	0.026	0.015	0.097
9	I’ve been getting help and advice from other people.	-0.051	0.133	-0.128	0.051	-0.026	0.042	0.271	0.109
10	I’ve been using alcohol or other drugs to help me get through it.	0.337	-0.103	-0.065	-0.038	-0.001	-0.049	-0.031	0.010
11	I’ve been trying to see it in a different light, to make it seem more positive.	-0.014	0.000	-0.029	-0.024	**0.515**	0.001	0.007	0.029
12	I’ve been criticizing myself.	-0.032	-0.049	-0.009	-0.0967	-0.012	0.046	**0.528**	-0.047
13	I’ve been trying to come up with a strategy about what to do.	0.047	0.017	0.078	-0.051	-0.036	0.0389	-0.200	**0.454**
14	I’ve been focusing on the good things in my life.	0.044	0.106	0.011	0.102	-0.037	-0.001	-0.004	-0.140
15	I’ve been praying or meditating.	0.281	-0.033	0.016	-0.073	0.005	-0.044	-0.053	-0.013
16	I’ve been making jokes about it.	-0.012	-0.079	**0.397**	-0.122	0.002	-0.031	0.017	0.040
17	I’ve been doing something to think about it less, like daydreaming, sleeping, or watching TV.	-0.060	-0.076	0.010	0.024	0.010	-0.001	**0.502**	-0.019
18	I’ve been accepting the reality of the fact that it has happened.	-0.095	0.390	-0.131	-0.100	-0.056	0.017	0.032	0.062
19	I’ve been expressing my negative feelings.	0.121	0.031	-0.104	0.068	0.050	-0.050	0.060	0.022
20	I’ve been trying to find comfort in my religion or spiritual beliefs.	0.022	0.193	0.068	0.026	-0.007	0.034	-0.072	-0.005
21	I’ve been trying to get advice or help from other people about what to do.	-0.009	-0.110	-0.020	0.002	0.027	-0.052	0.146	**0.428**
22	I’ve been learning to live with it.	-0.021	-0.016	0.038	-0.025	-0.014	**0.520**	0.066	-0.004
23	I’ve been thinking hard about what steps to take.	0.311	-0.042	-0.038	-0.076	-0.080	0.118	-0.056	-0.022
24	I’ve been blaming myself for things that happened.	-0.021	0.237	0.134	0.010	0.017	-0.008	-0.183	-0.053
25	I’ve been taking action to try to make the situation better.	0.070	0.084	0.134	0.042	-0.005	0.015	-0.045	-0.137
26	I’ve been making fun of the situation.	-0.038	0.081	-0.060	0.003	0.004	0.000	-0.011	**0.458**
27	I’ve been saying “This is not happening.”	-0.037	0.027	0.019	0.014	0.004	**0.517**	-0.002	-0.013
28	I’ve been blaming others for what happened.	-0.080	**0.400**	-0.132	-0.073	0.038	-0.035	-0.020	0.031

Exploratory factor analysis was conducted using principal axis factoring with varimax rotation. Factor loadings ≥ 0.40 are shown in bold and were considered salient. The Kaiser–Meyer–Olkin (KMO) measure of sampling adequacy and Bartlett’s test of sphericity supported the suitability of the data for factor analysis (*p* < 0.001). The eight-factor solution was retained based on eigenvalues > 1 and inspection of the scree plot

## Abbreviations

aOR: Adjusted odds ratio
BD: Bipolar disorder
Brief COPE: Brief Coping Orientation to Problems Experienced
CI: Confidence interval
cOR: Crude odds ratio
DSM-5: Diagnostic and Statistical Manual of Mental Disorders, Fifth Edition
DUREL: Duke University Religion Index
HIV: Human immunodeficiency virus
IR: Intrinsic religiosity
IQR: Interquartile range
KMO: Kaiser–Meyer–Olkin
LMICs: Low- and middle-income countries
MRRH: Mbarara Regional Referral Hospital
NORA: Non-organisational religious activity
ORA: Organisational religious activity
SD: Standard deviation
SSA: Sub-Saharan Africa
